# Nanomaterials for Functional Textiles and Fibers

**DOI:** 10.1186/s11671-015-1195-6

**Published:** 2015-12-29

**Authors:** Pedro J. Rivero, Aitor Urrutia, Javier Goicoechea, Francisco J. Arregui

**Affiliations:** Institute for Advanced Materials (InaMat), Materials Engineering Laboratory, Department of Mechanical, Energy and Materials Engineering, Public University of Navarre, Campus Arrosadía S/N, 31006 Pamplona, Spain; Institute of Smart Cities (ISC), Nanostructured Optical Devices Laboratory, Department of Electrical and Electronic Engineering, Public University of Navarre, Campus Arrosadía S/N, 31006 Pamplona, Spain

**Keywords:** Nanoparticles, Coatings, Functional textiles, Functional nanowebs, Electrospinning, Antibacterial, Flame retardant, UV-protection, Superhydrophobic, Membranes, Wound dressing

## Abstract

Nanoparticles are very interesting because of their surface properties, different from bulk materials. Such properties make possible to endow ordinary products with new functionalities. Their relatively low cost with respect to other nano-additives make them a promising choice for industrial mass-production systems. Nanoparticles of different kind of materials such as silver, titania, and zinc oxide have been used in the functionalization of fibers and fabrics achieving significantly improved products with new macroscopic properties. This article reviews the most relevant approaches for incorporating such nanoparticles into synthetic fibers used traditionally in the textile industry allowing to give a solution to traditional problems for textiles such as the microorganism growth onto fibers, flammability, robustness against ultraviolet radiation, and many others. In addition, the incorporation of such nanoparticles into special ultrathin fibers is also analyzed. In this field, electrospinning is a very promising technique that allows the fabrication of ultrathin fiber mats with an extraordinary control of their structure and properties, being an ideal alternative for applications such as wound healing or even functional membranes.

## Review

### Introduction

Natural fibers and textiles have been used for humans since ancient times. Our antecessors firstly used fur and animal skin for dressing and protection from the environment, but very soon they started to use vegetal fibers to make rudimentary fabrics. There are evidences of the use of dyed flax fibers into clothes more than 30,000 years ago [1]. For centuries, humans have used vegetal fibers (such as flax or cotton) and animal fibers (such as wool or silk) to produce yarns and then weave them into textiles using handmade processes. In the eighteenth century, there was a revolutionary industrial development [2] with the invention of machines that speeded up the manufacture of fabrics and made them more available and affordable. This technological revolution changed the concept of textile manufacturing transforming it into a real industry. It could be said that in the twentieth century, there was a second technological revolution with the synthesis of artificial fibers such as rayon [3], nylon, or polyester with good quality and low cost production techniques that rapidly gave those fibers a significant market share because of their good properties such as low cost, chemical stability, and outstanding versatility (dyes, colors, fiber diameters, engineered weaving for special clothes).

Nowadays, there is a new revolution on the textile industry with the apparition of new technologies that could add special functions and properties to the fabrics. For example, there has been significant improvement in technologies for textile coloring [1], digital printing on textiles [4], smart fabrics [5], and high performance functional textiles [6]. In this sense, nanoparticles play a key and significant role in this technological evolution since they show outstanding surface properties that allow to multiply their effect in comparison with bulky traditional additives and materials.

This work reviews the most relevant contributions of the use of nanoparticles for functionalize textile materials. Firstly, the analysis is focused on traditional synthetic fibers (mainly nylon, polyamide, or blends) and the use of nanoparticles for providing new properties such as antibacterial activity, flame retardant properties, UV-protection, supehydrophobicity, and others. And secondly, the use of nanoparticles is analyzed for special ultrathin fibers functionalization. These fibers can be obtained by electrospinnig technique for applications such as wound dressing, functional membranes for water treatment and others.

### Traditional Synthetic Fibers

The continuous advances in organic chemistry, especially with the apparition of polymer chemistry discipline, enable the fabrication of synthetic fibers which are made from polymers or small molecules, being the petroleum as the most important raw material. Among the high amount of different types of synthetic fibers, the most known are polyamide, polyester, acrylic, and polyolefin. Within these four types, the polyamide (especially PA6, known as polyamide 6 or nylon) and polyesters (especially PET, known as polyethylene terephthalate) are the most used in the textile industry. This section is focused on the development of functionalized synthetic fibers, concretely polyamide and polyester fabrics. In general, these fabrics show poor wettability properties and hydrophobicity in nature which can affect the polymer processability and also fiber dyebility [7]. With the development of the nanotechnology, different types of nanoparticles (mostly inorganic or metal oxide) have been successfully incorporated into synthetic fibers in order to obtain functionalized fibers with special properties [8–11]. The major effects such as flame retardancy, antibacterial activity, or superhydrophobicity are presented in detail using different deposition techniques. In addition, special emphasis is oriented to the surface modification fabrics in order to improve the adhesion of the nanoparticles and obtain functionalized synthetic fibers with the desired properties. Finally, it also discussed the importance of controlling particles size as well as their corresponding shape because both phenomena are of vital importance to the final and desired function imparted to the fibers.

#### Flame Retardant Applications

Synthetic fibers such as polyamide or polyester are highly inflammable, and continuous investigations are carried out in the development of flame retardant products because the legislation requires the use of low flammability products in the industry [12]. In this context, many of the available systems for flame retardancy are composed of halogen-containing additives. However, new regulations are devoted to the use halogen-free compounds, particularly phosphorous-based systems, which are being used as a promising alternative as effective flame retardants in textile industry [13–15]. Recently, the incorporation of nanoparticles in the finishing fabrics as flame retardant seems to be a valid and interesting approach. The main advantage is that a low amount of them can be employed, although not all the nanoparticles available in the market can be used in the flame retardancy field [16, 17]. It has been corroborated that several key points have to be into consideration in order to obtain better results. Among them, the resultant morphology (shape and size), chemical nature, concentration, as well as their distribution as a function of the procedure employed onto textile fabrics constitute the main ingredients to be an efficient flame retardant system.

El-Hady et al. proposed a novel flame retardant approach based on the use of zinc oxide (ZnO) nanoparticles for their application to cellulosic fabrics (cotton polyester blend) [18]. A pad-dry-cure method was selected to incorporate the ZnO nanoparticles onto the fabrics. The use of two different polycarboxylic acids such as succinic acid (SA) or 1,2,3,4-butane tetarcarboxylic acid (BTCA) together with sodium hypophosphite (SHP) as catalyst have been employed for cross-linking fabrics. In this work, firstly, the effect of curing temperature was investigated, and it has been demonstrated that an increase of the curing temperature from 160 to 180 °C made possible the formation of new ester cross-linking between cellulose chains and polycarboxylic acids [19]. And secondly, the measuring of char yield was performed to study the influence of flame retardant. The results indicated that both SA and BTCA are effective agents in reducing flammability of treated fabrics in the presence of SHP. However, better experimental results were observed for BTCA in comparison with SA in reducing flammability. In addition, it has been demonstrated that by increasing the nano ZnO concentration (from 0.25 to 0.5 %) as well as BTCA and SHP concentrations led to decrease the fabric flammability.

**Fig. 1:**
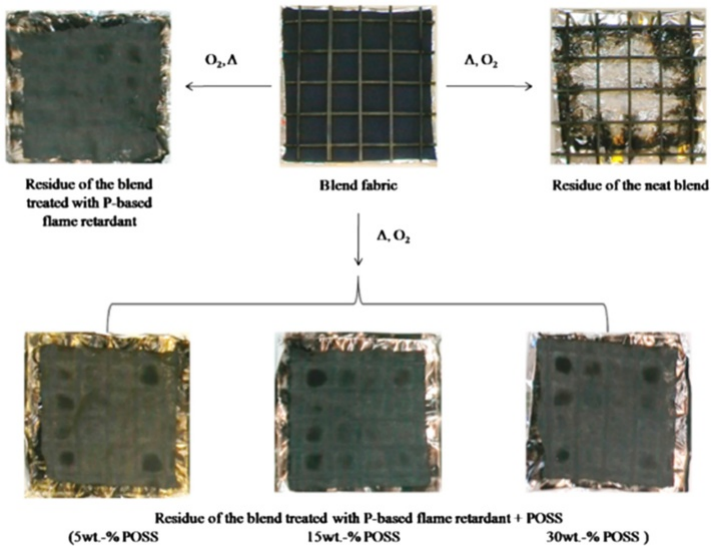
Residues of untreated PET-to-cotton blend and fabrics treated with a phosphorous-based flame retardant and variable globular nanoparticle (octalpropylammomium, POSS) amounts after cone calorimetry tests. Reprinted with permission from [16]

**Fig. 2:**
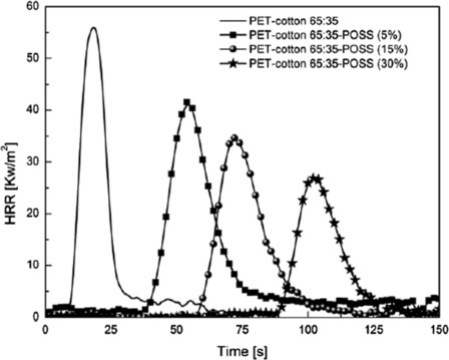
Heat release rate (HRR) curves of neat PET-to-cotton blend and of fabrics treated with a phosphorous-based flame retardant with different POSS amounts. Reprinted with permission from [16]

Novel studies about the influence of nanoparticles adsorption as fire protection for fabrics was presented by Alongi et al. [13, 17, 20]. This research group has studied the enhancement of the flame retardant properties for synthetic fibers such as polyester or cotton/polyester blends. The effect of impregnating different types of nanoparticles onto textiles as lamellar nanoparticles (montmorillonites CNa, hydrotalcites HT, or bohemites OS1), globular nanoparticles (titanium dioxide, silica, or octalpropylammonium POSS), or a mixing of both globular/lamellar nanoparticles has been evaluated [16]. In this sense, according to the use of lamellar nanoparticles, the use of dispersing agents such as sodium polyacrylate (B500) or binders such as dicarboxylate ester (PES) and an initial pre-treatment of the fabric by cold oxygen plasma makes possible an increase in time ignition and a reduction of heat release rate. As far as globular nanoparticles are concerned, the use of a silicon-based structure soluble in water as octalpropylammonium (POSS) shows promising results as flame retardant with an important decrease of CO_2_ and CO production. More specifically, polyester fabrics were not burnt when a methane flame was applied, although these results were not observed for cotton or PET-to-cotton blends. According to this, an interesting approach is presented in Fig. 1, where the results of untreated fabrics and fabrics coated with a combination of a commercial phosphorous-based flame retardant with different POSS amounts after cone calorimetry tests are shown. The untreated fabrics are totally destroyed after combustion test, whereas the sample only treated with the phosphorous flame retardant consisted of a thin film. However, the fabrics treated with both phosphorous flame retardant and variable POSS amounts (5, 15, and 30 wt.%, respectively) showed a residue more coherent and compact as a function of the nanoparticle amount. In addition, by increasing the POSS amount, the resultant time to ignition (TTI) and the corresponding peak value of heat release rate (pkHRR) were decreased in a remarkable way as it can be observed in Fig. 2. In addition, this same research group proposed that among all the possible nanoparticles combinations, a mixing of HT lamellar with SiO_2_ globular nanoparticles seemed to be the most efficient flame retardant system [21]. This aspect was corroborated using the fire performance index (FPI) which is calculated as TTI-to-pkHRR ratio. When a higher value of FPI is obtained, better results as flame retardant were observed. The highest improvements in fire performance were observed for the HT-SiO_2_ combination as evidenced all FPI values. These results were also corroborated for the PET-to-cotton blend where the HT-silica combination improves the fire performance to this blend. As a conclusion of the experimental results, a combination of both lamellar/globular nanoparticles presented better results as efficient flame retardant system in comparison with a single type of nanoparticles [16].

Until now, pad-dry-cure method or impregnation processes have been the most used techniques for the finishing fibers. However, an alternative approach for the incorporation of the nanoparticles into thin films is the layer-by-layer (LbL) assembly. This method makes possible the fabrication of nanocoatings onto the textiles fibers with a good control over the resultant thickness and roughness [22]. This method is performed by sequentially exposing the fabrics to a cationic polyelectrolyte and an anionic polyelectrolyte during a specific period of immersion time. In addition, a rinsing step in deionized water is necessary between both polyelectrolytes baths, and a drying step is performed between each rinsing time [20, 23, 24]. It is important to remark that the times of immersion as well as the times necessary for the rinsing and drying steps vary from author to author, although the methodology is always the same. According to this, Apaydin et al. designed new intumescent flame retardant coatings made from both cationic and anionic polyelectrolytes mixed with titanium dioxide (TiO_2_) nanoparticles using the LbL assembly [25]. These nanocoatings have been performed onto both polyamide and polyesters fabrics, showing significant differences in surface properties (i.e., wettability and surface energy) as it was corroborated by SEM images, EDX, and contact angle values. The resultant thin films covered homogeneously all external polyamide fabrics, whereas polyester fabrics were covered partially with the presence of aggregates. This important difference in morphology is the key point to understand the different behaviors to flammability tests and the reason that the coating only acts efficiently in reducing the peak heat release rate (pHRR) value for the polyamide fabrics. The incorporation of these TiO_2_ nanoparticles into LbL films slightly improved the fire retardant in comparison with a previous work of this research group based on polymeric coatings without any kind of nanoparticles [23].

Carosio et al. coated polyester (PET) fabrics by LbL assembly using negative colloidal SiO_2_ nanoparticles of two different sizes (10 and 30 nm, respectively) with positively charged alumina-coated SiO_2_ [20]. The best results have been observed for the smallest nanoparticles which increased the time to ignition and reduced the pHRR using cone calorimetry tests. One of the most relevant and important aspects of this work was the design of an environmentally friendly nanocoating based on nanoparticles, showing very promising results of flammability.

Finally, Conzalves et al. reported that multi-walled carbon nanotubes can be also used for improving the flame retardant properties when are applied onto polyester fabrics [26]. For this specific case, polyester fabrics functionalized with carbon nanotubes halved the burning rate and duplicated the burning time in comparison with raw polyester fabrics.

Finally, in order to have a better understanding of the different methodologies for obtaining flame retardant surfaces as well as the corresponding nanoparticles used for this purpose, a summary is shown in Table 1.

**Table 1:** Summary of the different types of nanoparticles with their corresponding size or the resultant thickness films for flame retardant applications as well as the deposition techniques used for a specific type of fabrics

Type of fabrics	Deposition process	Type of nanoparticles
Polyester and cotton/polyester blend	Impregnation process	Globular octalpropylammonium (POSS) nanoparticles, 2015 [16]
Cotton/polyester blend	Pad-dry-cure method	Zinc oxide (ZnO) nanoparticles (an average particle size of 30 nm), 2013 [18]
Polyester	Layer-by-layer assembly	Silica (SiO_2_) colloidal nanoparticles (<10 nm average thickness), 2011 [20]
Polyester and cotton/polyester blend	Impregnation process	Mixture of silica (SiO_2_) globular (spherical particles with an average size of 150 nm) and hydrotalcite (HT) lamellar nanoparticles, 2012 [21]
Polyamide and polyester	Layer-by-layer assembly	Titanium dioxide (TiO_2_) nanoparticles, thickness film (approx. 500 nm), 2015 [25]
Polyester	Layer-by-layer assembly	Multi-walled carbon nanotubes (MWCNTs) (an average diameter of 9.5 nm and an average length of 1.5 μm), 2012 [26]

#### Antibacterial Properties

Considering the special advantages of the application of nanostructured materials in textile industry, this section is focused on the modification of textile surface using different kinds of nanostructured materials which show excellent antimicrobial properties. Among all metallic materials, silver is of particular interest because it is a powerful antibacterial agent, showing antimicrobial efficacy against bacteria, viruses, and microorganisms [27, 28]. This excellent antibacterial activity makes possible their use in different and varied fields such as food preservation [29], safe cosmetics [30], medical devices [31, 32], water treatment [33], or textiles fabrics [34, 35]. An important aspect is that silver in both ionic and colloidal forms shows low toxicity to human cells and high biocompatibility [36]. However, the real mechanism of antimicrobial action related to the silver is not well understood, and different hypotheses are considered by the scientific community. Several studies indicate that the antibacterial effect is due to the release of silver ions from silver nanoparticles (Ag NPs), whereas other approaches are devoted to the adverse effect of the Ag NPs in the cell membrane, being these Ag NPs the responsible of the cell death. More details about these approaches can be found in [37].

As an example of multiple properties and their implementation in textile fabrics, Gerber et al. evaluated the effect of silver-tricalcium phosphate nanoparticles (Ag/TCP) onto polyamide 6 (PA6) fibers to build a reactive system against bacteria such as *Escherichia coli* and *Streptococcus sanguinis*, showing a killing efficiency of 99.99 or 100 % within 24 h contact time, respectively [38]. These great reactive properties can be implemented as self-disinfecting fibers in a broad range of applications. Other interesting approach is presented by Shastri et al. which evaluate the antimicrobial activity of nanosilver-coated sock fabrics against foot pathogens [39]. With the aim of the development of nanostructured silver production into fibers, Jiang et al. presented an effective method for preparing polyester fabrics via magnetron sputtering, showing excellent protection from ultraviolet protection, hydrophobicity, and good antibacterial performance [40]. SEM observations confirmed the presence of a thin layer of nanoscaled silver coating onto fiber surface after sputtering treatment. In addition, with an increase in sputtering time from 10 to 30 min, homogeneous deposition of silver nanoparticles (Ag NPs) with a particle size about 50 nm on the polyester fabrics is observed. In addition, the properties of the surfaces drastically change the resultant contact angle (CA) from hydrophilic (CA = 0°; untreated fabrics) to hydrophobic (CA = 132.2°) after modification with nanostructured silver. The silver-coated polyester has been tested against two kinds of bacteria such as *E. coli* and *Staphylococcus aureus*, showing an excellent antibacterial reduction ratio of 99.7 and 99.8 %, respectively. Interesting approaches were presented by Radetic et al. [41–43], which are devoted to the use of a corona treatment (electrical discharge at atmospheric pressure) or low-temperature radio frequency (RF) plasma for fiber surface activation which can facilitate the binding efficiency of colloidal Ag NPs onto polyester as well as polyamide fabrics, showing an enhancement of the antibacterial properties and better laundering durability.

**Fig. 3:**
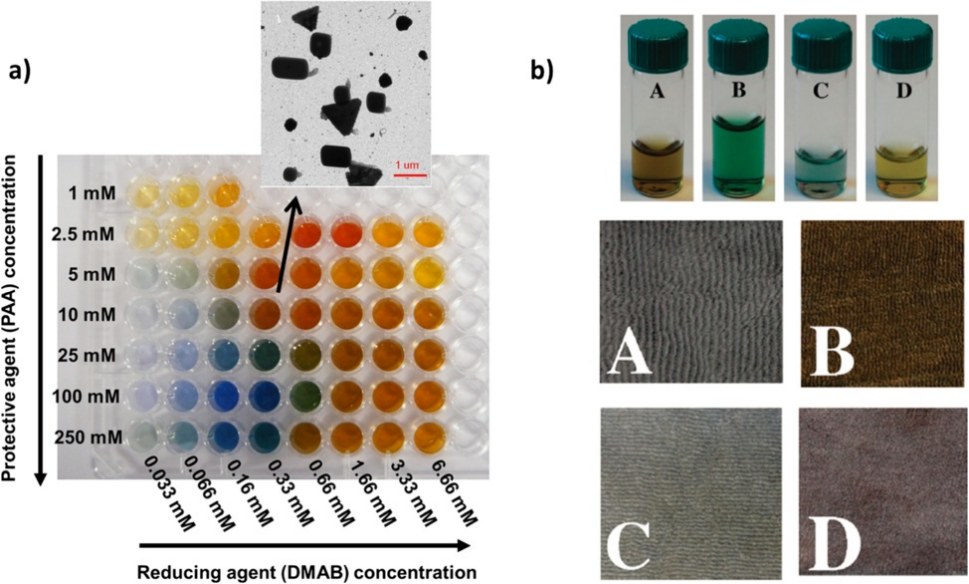
**a** Photograph of multicolor silver map obtained as function of variable protective agent and reducing agents. TEM micrograph shows the formation of Ag NPs with different shapes (*triangle*, *rod*, *cube*, *bar*) for 10 mM PAA and 0.33 mM of DMAB. Reprinted with permission from [45]. Copyright (2013) Springer. **b** Silver nanosols prepared at pH 8 and silver-nanosol-loaded polyester fabrics (*A*) NaBH4 reduction, *M*_w_ 1200, (*B*) NaBH4 reduction, *M*_w_ 15,000, (*C*) UV reduction *M*_w_ 1200, (*D*) UV reduction *M*_w_ 15,000. Reprinted with permission from [46]. Copyright (2008) American Chemical Society

As an important aspect to be in consideration is that there is a wide variety of synthetic methodologies to obtain Ag NPs. Most of them are focused on a strict control of several parameters such as shape, size, surface functionalization, or interparticle distance which affect their final properties [44]. Rivero et al. designed an experimental matrix where multicolored Ag NPs (Fig. 3a) were obtained as a function of the fine control of two parameters such as protective agent (polyacrylate (PAA)) and reducing agent (dimethylaminoborane (DMAB)) concentrations [45]. A great control of these parameters makes possible the synthesis of Ag NPs or silver clusters with different shapes and sizes.

**Fig. 4:**
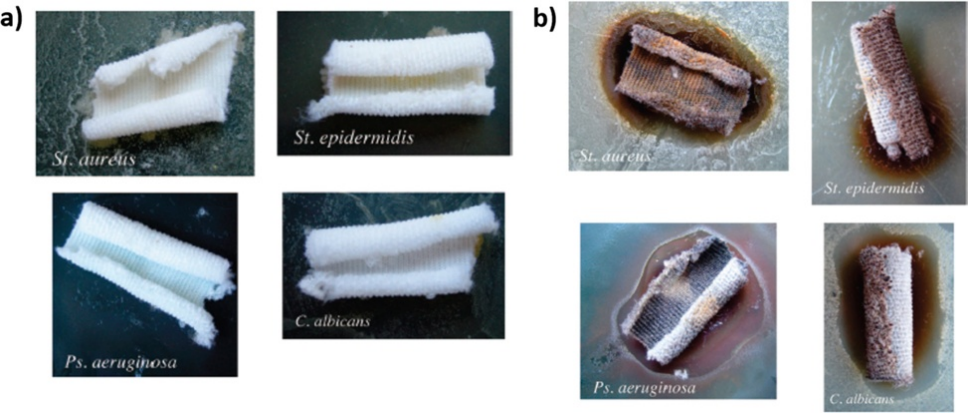
**a** Effect of untreated polyester samples toward the growth of the investigated stains; **b** effect of Ag-treated polyester samples toward the growth of the investigated stains. Reprinted with permission from [46]. Copyright (2008) American Chemical Society

In this sense, based on the preparation of silver-poly(acrylates) clusters, Falleta et al. proposed the synthesis of silver nanosols using silver nitrate (AgNO_3_) as a source of silver ions, PAA as a protective agent at two different molecular weights (*M*_w_ 1200 and *M*_w_ 15,000) and two different reducing mechanisms (chemical reduction with sodium borohydride, NaBH_4_ and UV radiation exposure), showing all dispersions different colorations (Fig. 3b, top) [46]. UV-vis spectra corroborated the presence of well-separated absorption bands. One was located at 400 nm which is related to the localized surface plasmon resonance (LSPR) absorption band of the silver, whilst another band with a broad signal was located at visible region which is associated with the formation of silver-polyacrylate complexes. These nanosols were applied onto polyester textiles by simply immersing, and the treated fabrics maintained the coloration after the rinsing and drying steps which corroborate the complete adhesion of the nanoparticles onto the fabrics surface (Fig. 3b, down). The resultant-treated polyester fabrics showed a strong inhibition effect on all microorganisms (*S. aureus, Staphylococcus epidermidis, Pseudomonas aeruginosa*, and *Candida albicans*) whereas the untreated fabrics did not show any inhibition zone, as it can be observed in Fig. 4.

Other alternative approach is the synthesis of nanosilver on fabrics using silver ammonia complex [47–49]. Textor et al. proposed the modification of polyamide fabrics surface using a heterogeneous Tollens’ reaction [47]. In a first step, the surface was modified by treatment with glutardialdehyde (GDA), making possible the establishment of aldehydes at the polymer surface. In order to corroborate the presence of this aldehyde layer, measurements of the contact angle of water for GDA-treated polyamide films were performed. An increase of contact angle from 50° (untreated) to 66° (the highest GDA concentration) verified the surface modification. Further, in a second step, polyamide fabrics were treated with Tollens’ reagent, making possible the formation of a thin silver layer due to the chemical reduction of silver ions (Ag^+^) to Ag NPs for the presence of aldehydes. A color change to brownish indicated a complete reduction of Ag^+^ in solution, and it is assigned to the surface plasmon resonance of silver layers of a thickness in the range of several nanometers [45]. The experimental results showed an excellent antimicrobial activity of the silver finished polyamide fabrics against *E. coli* and even more an excellent durability of the coated fabrics even after 30 laundry cycles.

A similar approach based on the use of silver-ammonia complex is presented by Montazer et al. [48]. In this case, Ag NPs have been prepared using polyvinyl pyrrolidone (PVP) as a reducing/stabilizing agent through UV irradiation which reduce silver ions (Ag^+^) to nanoparticles (Ag^0^). These nanoparticles have been deposited onto nylon-knitted fabric surface using a simple dip-pad technique. The antibacterial activity of the treated fabrics has been tested against *E. coli* and *S. aureus*, showing a reduction percentage of the bacteria growth up to 99.2 % after 20 washes. This same research group proposed later the synthesis of Ag NPs within the polymeric chains of PA6 fabrics by using a silver-ammonia complex [Ag(NH_3_)_2_]^+^ [49]. It was demonstrated that the polymeric chains of polyamide acted as both stabilizing and reducing agents for the in situ synthesis of the nanoparticles without using any other type of chemical compounds as stabilizing or reducing agents. The silver/ammonia complex could be oxidized through the polyamide chains, and consequently, the silver ions could be reduced to Ag NPs. Furthermore, the nitrogen atoms precedent of polyamide chains can stabilize Ag NPs through coordination. Due to this fixation of Ag NPs within polymeric chains of polyamide, the resultant-coated fabrics showed excellent antibacterial properties against *E. coli* and *S. aureus* even after repeated washing cycles.

Another different alternative method for the preparation of Ag NPs is the in situ synthesis process. The in situ synthesis of Ag NPs is based on chemical reactions in solution (often termed wet chemistry) that yields loading metal ions and a further synthesis of metal nanoparticles. The use of an adequate encapsulating and reducing agent is one of the keys to synthesize the metal nanoparticles with a specific morphology. In this contest, Babaahmadi et al. reported a novel and one-step method for the in situ synthesis of Ag NPs through reduction of silver nitrate (AgNO_3_) with stannous chloride (SnCl_2_) on polyamide (PA) fabrics, using cetyltrimethyl ammonium chloride (CTAB) as stabilizer [50]. The treated fabrics showed good antibacterial properties against both Gram-positive (*S. aureus*) and Gram-negative (*E. coli*) bacteria. The quantitative tests were performed based on the inhibition zone surrounding of the samples, as a function of inhibition or not bacterial growth. According to this same methodology, Allahyarzadeh et al. proposed the in situ synthesis of nanosilver on polyester fabrics using NaOH/Nano TiO_2_ with self-cleaning and antibacterial properties [51]. For this purpose, the alkali hydrolysis makes possible the enhancement of the surface activity, improving the nanoparticle absorption and producing ethylene glycol. The Fourier transform infrared (FTIR) spectra corroborated the presence of ethylene glycol because the intensity of characteristic peaks attributed to –OH at 3054 and 3419 cm^−1^ and the peak attributed to C=O (carboxylic acid) at 1716 cm^−1^ were increased, proving the production of hydroxyl (–OH) and carboxyl (–COOH) functional groups on the fiber surface. After that, the oxidation of hydroxyl groups to aldehyde groups in ethylene glycol resulted in carboxylate ions and electrons, making possible the final reduction of silver ions to Ag NPs. In addition, X-ray diffraction (XRD) confirmed the presence of Ag NPs on the fabric surface. The synthesis of these Ag NPs onto polyester fabrics resulted in excellent antibacterial efficiency against *S. aureus* and *E. coli*. This methodology of in situ synthesis can be also used for synthesizing copper nanoparticles within the polymeric chains of nylon fabrics by using ascorbic acid as a reducing agent and CTAB as a capping agent [52] as well as for synthesizing nanometal oxides in nonwoven polyester fabrics [53].

As it has been previously commented, not only Ag NPs show antibacterial activity. It has been demonstrated that nanometal oxides can be used as antibacterial agents such as titania (TiO_2_), silica (SiO_2_), or zinc oxide (ZnO) nanoparticles can be also used as an antibacterial finishing for textiles [54–56]. Among all these nanometal oxides, it is well-known that TiO_2_ nanoparticles show extraordinary photocatalytic activity, non-toxicity, high availability and biocompatibility, and being of great interest for manufacturing processes in order to impart the specific antibacterial properties. However, as it happened with Ag NPs, the exact mechanism of the bactericidal activity of the TiO_2_ nanoparticles is not well understood and several approaches are used to demonstrate the TiO_2_ action in the corresponding bacterial cell death [57].

Montazer et al. used an enzymatic pretreatment to improve the functionality of textile materials based on TiO_2_ nanoparticles, showing great results as self-cleaning, antibacterial, and UV-protection [54]. The methodology is based on two well separately steps. In a first step, a polyester/wool fabric as a blend of fibers fabric was treated with two different types of enzymes such as lipase or protease to hydrolyze the wool and polyester surface, respectively, increasing the surface activity. In a second step, the fabrics were dipped into an ultrasonic bath containing nano-TiO_2_ and a cross-linking agent, BTCA. This agent was used to enhance the nanoparticles adsorption onto the fabric surface. The treated samples exhibited excellent antibacterial activity against *E. coli* as the reduction of bacteria could reach to 100 % with 0.75 % nano-TiO_2_ and 99 % with 0.25 % nano-TiO_2_. In this contest, related to the use of TiO_2_ nanoparticles, Mihailovic et al. demonstrated the novel properties of polyester fabrics modified by corona discharge or RF plasma and colloidal TiO_2_ nanoparticles [58–60]. This specific treatment provided a better binding efficiency of the TiO_2_ nanoparticles onto the polyester fabrics. The results of the treated fabrics showed multifunctional properties such as antibacterial activity, self-cleaning effect with long-term stability, and good UV-protection.

El-Gabry et al. have treated polyester fabrics with SiO_2_ nanoparticles to improve the properties of textile fabrics [55], showing good antibacterial properties and excellent durability of the treatment to repeat home laundering, thanks to the use binder such as an acrylate copolymer.

Farouk et al. proposed the design of hybrid polymers based on nanosized ZnO particles, being a great alternative as an antibacterial finishing for textiles [56]. These hybrid materials are based on glycidiltrimethoxisilane (GPTMS) and were applied onto cotton/polyester (65/35 %) fabrics, using a padding process. The antimicrobial activity of the modified fabrics with a constant mass of ZnO (10 wt.%) but with a varying particle size was tested against Gram-negative bacteria (*E. coli*) and Gram-positive bacteria (*Micrococcus luteus*). An important consideration of this work is that the fabrics treated with the hybrid polymer loaded with ZnO particles of the smaller particle size (30–60 nm) leads to a complete annihilation of both bacteria, while fabrics coated with hybrid polymers loaded with bigger ZnO particles (600–650 nm) achieve a 93 and 97 % reduction of the *E. coli* and *M. luteus* populations, respectively. This enhanced bioactivity related to the smaller particles is associated with the higher surface-area-to-volume ratio.

Other approaches for obtaining antibacterial activity can be performed with the design of nanocomposites (Ag/ZnO, Ag/SiO_2_) [61, 62] or well with the combination of metal nanoparticles which show separately antibacterial properties [63, 64]. As it has been previously demonstrated, the use of ZnO nanoparticles with antimicrobial activity for textile treatment [56], this same research group proposed in a later paper the preparation of Ag/ZnO composite nanoparticles by the reduction of silver on the surface of ZnO nanoparticles [61]. The coating process was carried out on cotton/polyester (50/50 %) blend fabrics, and the antimicrobial tests were performed against the same kind of bacteria *E. coli* and *M. luteus*. As a conclusion of the results, the antibacterial activity of Ag/ZnO nanoparticles increases with a higher concentration of silver. In addition, this increasing of antimicrobial activity for the higher silver concentration in comparison with the other samples can be associated with decreased crystallite size with the corresponding increase of the active surface area, making possible an enhancement in the biocidal effect.

An interesting approach is presented by Shin et al. where silver-doped silica-complex nanoparticles have been synthesized for the design of antibacterial materials [62]. In this work, firstly, Ag NPs were synthesized using both liquid-phase and alcohol reduction methods, and secondly, silver-doped silica-complex nanoparticles were synthesized by using a sol-gel process, showing an excellent antibacterial activity against *S. aureus* and *E. coli*. As a conclusion obtained by silver-doped silica-complex nanoparticles, the average size of the nanoparticles decreased when the molecular weight (*M*_w_) of the stabilizing agent PVP. In addition, the number of particles increased by a mercapto treatment (–SH group) from mercaptopropyl trimethoxysilane (MPTMS) which showed a narrower size distribution in comparison with the silica treated by an amino treatment (–NH_2_ group) from aminopropyl trimethoxysilane (APTMS).

Ali et al. proposed the synthesis of both chitosan nanoparticles (CSN) and silver-loaded chitosan nanoparticles (Ag-CSN) and their further incorporation onto fabrics with the aim of designing bioactive polyesters [63]. An ionic gelation method using chitosan as a polycation and sodium tripolylphosphate (TPP) as a polyanion was used to prepare the CSN nanoparticles, whilst a further loading with silver ions was used to prepare the Ag-CSN nanoparticles. In addition, the minimum inhibitory concentration (MIC) against S*. aureus* bacteria was found to be 50 and 500 times less for CSN and Ag-CSN, respectively, as compared to bulk chitosan material. The Ag-CSN nanoparticles showed a further increase in activity due to the synergistic effect of Ag and chitosan nanoparticles. These results indicated that an antibacterial activity of 90 or 100 % were obtained for CSN and Ag-CSN nanoparticles, respectively, at very low concentration (0.2 %), whereas the bulk chitosan showed an antimicrobial activity of 58 % for the same concentration.

Another interesting approach based on the simultaneous combination of two different types of nanoparticles is presented by Mihailovic et al. [64]. In this work, it is shown the potential of combining both colloidal Ag and TiO_2_ NPs onto polyester fabrics, making possible to obtain multifunctional properties such as antimicrobial activity, UV protection, and photocatalytic behavior onto the fabrics surface. According to the antimicrobial tests, it is important to remark that the fabrics treated with both types of nanoparticles (TiO_2_ and Ag NPs) provided a higher antimicrobial efficiency in comparison with the fabrics treated only with Ag NPs. The modified fabrics were tested against Gram-negative bacteria (*E. coli*), Gram-positive bacteria (*S. aureus*), and fungus (*C. albicans*).

Mahltig et al. proposed the application of three different modified silica sols containing silver components onto polyamide fabrics, showing a lasting antimicrobial effect against *E. coli* even after 40 washing cycles [65]. The different sols (sol A, sol B, and sol C, respectively) were applied onto polyamide fabrics using a pad-dry-cure process. In order to determine the antimicrobial effect of the sol-gel coatings, the number of colony forming units was evaluated as the samples are prepared as well the number of washing cycles are increased up to 40 washes. It was observed that after coating preparation, the antimicrobial effect related to both sols A and B was lower in comparison with sol C. The main reason of this different behavior is related to the chemical composition of the sols. Although both sols A and B presented the same silver source (silver nitrate, AgNO_3_), the sol C presented a different one (commercial colloidal silver). In addition, both sols B and C consisted of a quaternary ammonium compound such as hexadecyltrimethylammonium-p-toluenesulfonate (HTAT) used as a dispersing/stabilizing agent which is also well-known because of its antimicrobial activity. Due to this, sols B and C showed better results due to the presence of HTAT in their chemical composition in comparison with sol A. However, a drastic change in their antimicrobial effect was observed after 40 washing cycles because the polyamide fabrics coated with sol C lost all their antimicrobial activity. This important difference could be associated with the silver source in sol C (commercial colloidal silver) which was not so tightly fixed to the fabric surface. SEM images of the coatings showed particles of bigger size (micrometer range) with a flake shape for polyamide fabrics coated with sol C whereas particles of smaller size (below 100 nm) with a spherical shape were observed for fabrics coated with sols A and B. Due to this, the particles of bigger size are easily abraded under conditions of repeated washing cycles as it happens for sol C. When sol A with sol B was compared, smaller and more regular distribution of the in situ synthesized nanoparticles onto polyamide fabrics were observed for sol B, due to the use of a surfactant such as HTAT, and it is the reason of their higher durability under the conditions of repeated washing cycles with a better antimicrobial efficiency after 40 cycles.

It is important to consider that there are other types of synthetic fibers such polypropylene (PP) or polypropylene/polyethylene (PP/PE) which can be also modified to show antibacterial activity [66, 67]. As an example, it has been corroborated the antibacterial efficacy onto nonwoven polypropylene using gold nanoparticles [66] or well onto padded PP/PE nonwoven by using different nanosilver colloids [67].

Finally, a summary is shown in Table 2 of the different nanoparticles used for antibacterial activity and the possibility of obtaining high durability antimicrobial effect after successive washing cycles. As it has been previously commented, the vital importance of controlling the corresponding size of the nanoparticles plays a key role in the resultant antibacterial activity of the nanoparticles [56, 61, 63, 65]. A decrease in the corresponding size show better results in the antibacterial tests due to the higher surface-area-to-volume ratio in comparison with bulky materials [63] or particles of bigger size [65]. In addition, Table 2 also summarizes the fabrics which show laundering durability as a function of an adequate surfactant or a specific pretreatment to enhance the adhesion of the nanoparticles onto the fabrics surface.

**Table 2:** Summary of the type of nanoparticles used for antibacterial or antifungal activity

Antibacterial agent	Fabrics	Antibacterial or antifungal tests
Silver-tricalcium phosphate NPs (Ag/TCP)	Polyamide	*E. coli* and *S. sanguinis*, 2011 [38]
Silver nanoparticles	Polyester	*E. coli* and *S. aureus*, 2010 [40]
Silver nanoparticles	Polyester and polyamide	*E. coli* and *S. aureus*; laundering durability pretreatment: corona, 2008 [41]
Silver nanoparticles	Polyester and polyamide	*C. albicans*; laundering durability pretreatment: corona, 2009 [42]
Silver nanoparticles	Polyester	*E. coli* and *S. aureus*; laundering durability pretreatment: radio frequency (RF) plasma, 2010 [43]
Silver nanoparticles	Polyester	*S. aureus, S. epidermidis, P. aeruginosa*, and *C. albicans*, 2008 [46]
Silver ammonia complex	Polyamide	*E. coli;* laundering durability pretreatment: glutaraldehyde (GDA), 2010 [47]
Silver ammonia complex	Polyamide	*E. coli* and *S. aureus*, 2012 [48]
Silver ammonia complex	Polyamide	*E. coli* and *S. aureus*; laundering durability, 2014 [49]
Silver nanoparticles	Polyamide	*E. coli* and *S. aureus*, 2015 [50]
Silver nanoparticles (in situ synthesis)	Polyester	*E. coli* and *S. aureus*, 2013 [51]
Copper nanoparticles (in situ synthesis)	Polyamide	*S. aureus*, 2013 [52]
TiO_2_ nanoparticles	Polyester/wool	*E. coli*, 2011 [54]
TiO_2_ nanoparticles	Polyester	*E. coli* pretreatment: oxygen and argon plasma, 2010 [58]
TiO_2_ nanoparticles	Polyester	*E. coli* Pretreatment: corona/air RF plasma, 2011 [59]
SiO_2_ nanoparticles	Polyester	*E. coli* and *S. aureus*; laundering durability binder: acrylate polymer, 2013 [55]
ZnO nanoparticles	Cotton/polyester	*E. coli* and *M. luteus*, 2014 [56]
Ag/ZnO composite nanoparticles	Cotton/polyester	*E. coli* and *M. luteus*, 2014 [61]
Silver-doped silica-complex nanoparticles	Polyester	*E. coli* and *S. aureus*, 2014 [62]
Chitosan and silver-loaded chitosan nanoparticles	Polyester	*S. aureus*, 2011 [63]
Mixture of silver and TiO_2_ nanoparticles	Polyester	*E. coli*, *S. aureus* and *C. albicans*, 2011 [64]
Silica sols with silver nanoparticles	Polyamide	*E. coli;* laundering durability, 2010 [65]
Gold nanoparticles	Polypropylene	*E. coli* and *S. aureus*; laundering durability pretreatment: air plasma treatment, 2013 [66]
Silver nanoparticles	Polypropylene/polyethylene	*E. coli, Klebsiella pneumoniae*, and *S. aureus*, 2005 [67]

#### Superhydrophobic Surfaces

The fabrication of superhydrophobic materials with oil/water repellent properties has been receiving great attention in the textile industry [68]. The concept of superhydrophobicity can be defined as how those surfaces which present water contact angles higher than 150°, which is also commonly known as the artificial lotus effect. If the same contact angle (always higher than 150°) is obtained with oil, the surface is said to be superoleophobic [69]. Nowadays, there is a wide variety of methodologies which are based on the combination of a specific topography and an adequate chemical compound to produce a high contact angles [70, 71]. Theoretical models are based on both low surface free energy and high degree of roughness. Due to this, the superhydrophobicity depends strongly on the surface chemistry and the surface morphology [72]. Many approaches for building a superhydrophobic surface consist of a nanostructured surface and followed by a treatment with a fluoro-containing polymer or silane [73, 74]. A clear example onto polyester textile fabrics is presented by [75]. In this case, an alkaline hydrolysis plays a key role in the possible surface roughening effect and is applied to manipulate the surface morphology, whilst a further fluorocarbon layer is deposited in order to generate low surface energy. Other approaches based on the same methodology are presented by [69, 76]. Saraf et al. explored different techniques such as pulse plasma polymerization of perfluorodecyl acrylate (PFAC8), microwave-assisted fluorosioxane condensation, or fluorosiloxane condensation via wet processing in order to achieve superhydrophobicity and superoleophobicity on nylon nonwoven fabrics [69]. The treated fabrics showed very high contact angles for both water (168°–174°) and dodecane (153°–160°). Zhu et al. designed a superhydrophobic polyester fabric with a good mechanical stability and an easy repairability by coating the fabric with metallic Ag NPs and a further surface fluorination [76]. The superhydrophobic properties were maintained after immersion test, finger pressing, or abrasion with sandpaper, showing a high superhydrophobic permanency. However, when a loss of superhydrophobicity occurs, it is possible to regenerate the fabric surface by an easy repair process which consists of repeating silver deposition and surface fluorination process.

Alternative new approaches for the design of superhydrophobic surfaces in the textile industry are focused on the surface functionalization of SiO_2_ nanoparticles with non-fluorinated alkylsilanes [77] or fluorosilanes [78–80]. As an example of the use of these functionalized SiO_2_ nanoparticles, an interesting approach for imparting extremely high hydrophobicity to cotton and polyester fabrics is presented by Gao et al. [77]. For this case, a first step is based on the fabrication of a silica sol prepared by the respective hydrolysis and condensation of tetraethyl ortosilicate (TEOS) under alkaline conditions. Then, a second hydrophobization step was performed using hydrolized hexadecyltrimethoxysilane (HDMTS). The treated cotton and polyester textiles show excellent water repellent properties with a water contact angle of 155° on cotton and 143° on polyester. This high hydrophobicity is associated with the presence of a non-fluorinated compound such as HDMTS as well as an increase in roughness by the silica sol. An important aspect was the high durability of the treated textile fabrics because they were able to retain the most of the hydrophobic properties after 30 laundering cycles.

Xu and coworkers designed fluoropolymer/silica (FP/SiO_2_) organic/inorganic nanocomposites coatings on polyester fabrics [78, 79]. For the preparation of these nanocomposites, firstly, vinyl silica (V-SiO_2_) hydrosols were prepared by one-step water-based sol-gel method using the vinyl trimethoxy silane (VTMS) as the silane precursor and ammonium hydroxide as the catalyst. Secondly, a fluorinated acrylic polymer/silica nanocomposite was prepared by emulsion polymerization based on V-SiO_2_ hydrosol. FTIR measurements corroborate that the V-SiO_2_ nanoparticles had been successfully incorporated into the nanocomposite and the fluoropolymer had been chemically bonded with nano-SiO_2_ by the vinyl bridge. A dip-pad-cure process was applied to deposit the nanocomposite onto the polyester fabrics. It has been demonstrated that the treated fabrics with these fluoropolymer/SiO_2_ nanocomposites showed higher contact angle values than the fabrics which were only treated with a fluoropolymer. The nanocomposites exhibited excellent superhydrophobicity with a contact angle of 151.5° and a water shedding angle of 12°, whereas the polyester fabric treated by fluoropolymer without the presence of V-SiO_2_ nanoparticles showed a contact angle of 132.3° and a water shedding angle of 29°. These results corroborate the importance of a rough surface structure on PET fabrics which is provided by the SiO_2_ nanoparticles to improve the hydrophobic properties of the fluorinated polymers which provide low surface energy.

**Fig. 5:**
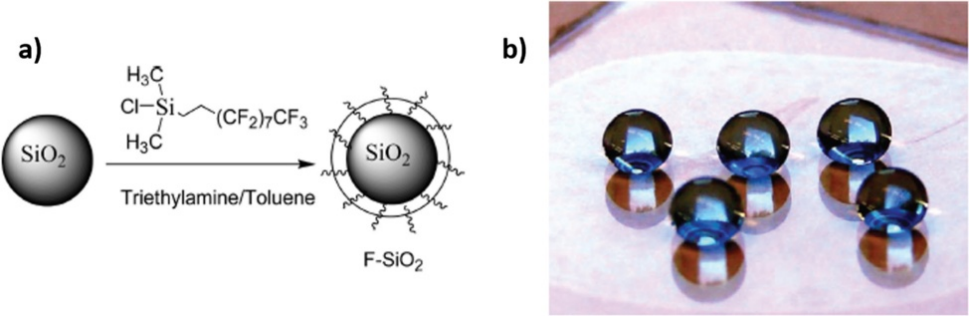
**a** Schematic illustration of the synthesis of fluorosilane-coated silica nanoparticles, F-SiO_2_ NP; **b** optical photograph of water droplets (>10 μL) on F-SiO2 NP-coated polyester fabric. A small amount of dimethyl methylene blue dye was dissolved in water for illustration purpose. Reprinted with permission from [80]. Copyright (2012) American Chemical Society

**Fig. 6:**
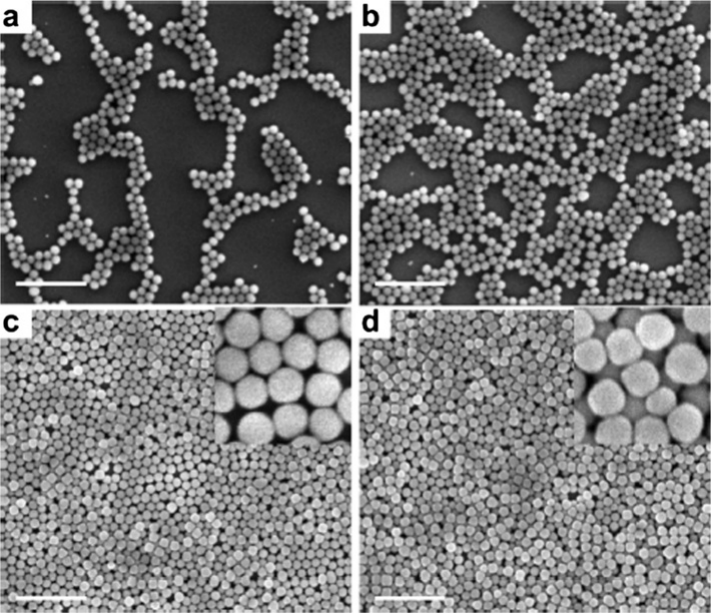
SEM images of spin-coated 100-nm F-SiO2 NPs with different concentrations on TESPSA-functionalized Si wafers: **a** 0.1, **b** 0.4, **c** 0.8, and **d** 1.2 wt.%. The *insets* in **c** and **d** are high-magnification images. Scale bars: 1 μm. Reprinted with permission from [80]. Copyright (2012) American Chemical Society

A novel approach for obtaining transparent and superhydrophobic surfaces by spin-coating technique is reported by Xu et al. using fluorosilane-functionalized SiO_2_ nanoparticles (F-SiO_2_ NP) [80]. In this case, firstly, commercial SiO_2_ nanoparticle (100 nm in diameter) surfaces were functionalized with a fluorosilane in the presence of triethyl amine (TEA) and toluene (Fig. 5a). And secondly, these hydrophobic nanoparticles were applied onto the polyester surface using the spin-coating technique in a fluorinated solvent (Fig. 5b). The treated polyester fabrics exhibited superhydrophobicity with an advancing water contact angle greater than 150° and a water droplet roll-off angle less than 5°. In addition, it is important to remark the high optical transparency of the spin-coated films, showing greater than 95 % transmittance in the visible region. An important consideration is that SEM images reveal that by increasing the F-SiO_2_ NP concentration, the assembly of the nanoparticles onto the substrate surface changed from random and nonclose packed (0.1 or 0.4 wt.%) to nearly close-packed (≥0.8 wt%) (Fig. 6). Due to this close-packed configuration, the spin-coated surface became superhydrophobic with an advancing water contact angle higher than 150°.

Other different approaches for obtaining superhydrophobic synthetic fabrics are based on the incorporation of ZnO particles using different techniques [81–83]. For example, Fruza et al. reported that polyester fabrics were successfully coated with crystalline particles of ZnO using an electroless deposition [81]. Morphological analysis obtained by XRD and SEM indicated the formation of hexagonal prism crystallites (wurtzite type), having a variable size between 20 and 500 nm in diameter and up to 1 μm in length. The most important aspect of the incorporation of these ZnO prisms onto surface fabrics is the change from a hydrophobic surface (raw polyester fabric) to superhydrophobic surface because the water contact angles exceeded 150°.

Other alternative method to obtain superhydrophobic polyester fabrics is presented by Ashraf et al. [82]. In this specific work, ZnO particles with a nanorod shape were grown by seeding method with the aim to develop hierarchical roughness structure. The presence of the nanorods onto surface fabrics was corroborated by XRD analysis where three sharp peaks indicated a good crystallinity of the ZnO particles. Once the nanorods have been successfully incorporated on polyester fabrics, a second step is based on a further modification of the fabrics with octadecyltrimethoxysilane (ODS). It is important to remark that a change of the surface properties is observed in the PET fabrics from a previous treatment with ODS to PET treated with ODS. Before modification with ODS, the fabrics showed superhydrophilicity and water contact angle decreased as the seed concentration growth. However, after ODS treatment, the fabrics drastically changed their behavior, showing a lotus effect with very high values of water contact angle.

This same change in the surface properties is reported by Popescu et al. [83]. In this work, they proposed the functionalization of cotton/polyester woven fabrics with ZnO thin films or nanoparticles by pulsed laser deposition. It has been demonstrated that by changing the number of laser pulses, well-separated nanoparticles (ten pulses) or compact thin films (100 pulses) were obtained. In addition, the ZnO nanoparticles deposited onto fabrics can switch the wetting behavior of the textile from hydrophilic to hydrophobic as a function of ambient gas nature and the pressure in the deposition chamber. The best results were observed when it was deposited a thin film of ZnO in vacuum, showing superhydrophobicity with a water contact angle of 157°.

**Fig. 7:**
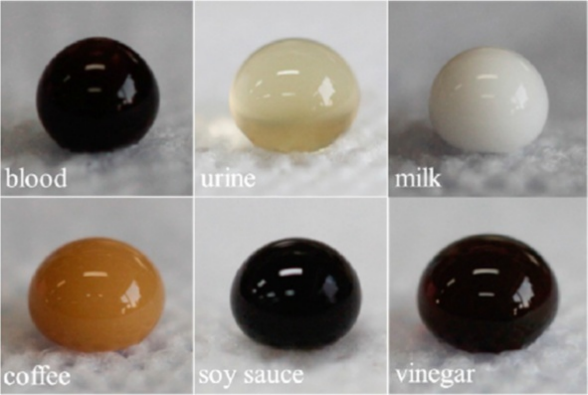
Digital photographs of various liquid droplets of about 80 μL in volume testing on PP nonwoven fabrics. Reprinted with permission from [85]. Copyright (2015) American Chemical Society

Finally, superhydrophobic surfaces onto polypropylene textiles can be also obtained [84, 85]. As an example, Zhu et al. proposed a simple, rapid, and scalable method to fabricate superhydrophobic polypropylene fabrics by solvent swelling method [85]. The key role of this work is that the fabrics surface is swelled by heated solvent (cyclohexane/heptane mixture) and then a shrinking of the polymeric chains is observed during the solvent evaporation process, leading the formation of submicron protuberances. As a result, the treated fabrics showed excellent repellence to various liquids such as blood, urine, milk, coffee, soy sauce, or vinegar (Fig. 7), showing a great durability and robustness.

Finally, the different methodologies for obtaining superhydrophobic surfaces as well as the corresponding nanoparticles or nanomaterial used for this purpose onto specific fabrics are summarized in Table 3.

**Table 3:** Summary of the type of nanoparticles or nanomaterials used for superhydrophobic surfaces

Type of fabrics	Deposition process	Superhydrophobic surface
Polyester	Pad-dry-cure method	Alkaline hydrolysis and fluorocarbon layer, 2011 [75]
Polyester	Dip-coating	Silver nanoparticles and fluorination, 2012 [76]
Polyester	Dip-pad-cure process	Silica (SiO_2_) nanoparticles and fluropolymer, 2015 and 2014 [78, 79]
Polyester	Spin-coating	Silica (SiO_2_) nanoparticles and fluorosilanization, 2012 [80]
Polyester	Electroless deposition	Zinc oxide (ZnO) nanoparticles, 2013 [81]
Polyester	Solution or vapor deposition	Zinc oxide (ZnO) nanoparticles and octadecyltrimethoxysilane (ODS), 2013 [82]
Cotton/polyester	Pulse laser deposition	Zinc oxide (ZnO) nanoparticles, 2011 [83]
Polypropylene	Solvent swelling method	Swollen of the polymeric chains, 2015 [85]

### Nanofibers and Nanowebs

**Fig. 8:**
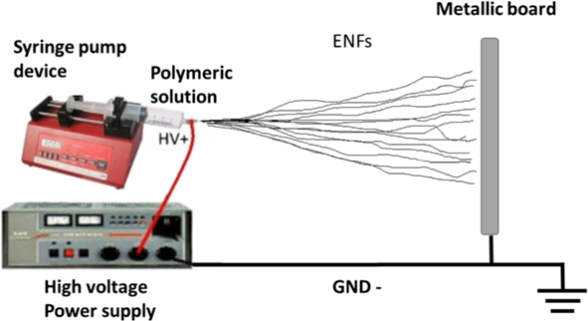
Setup scheme of the simple electrospinning technique to produce electrospun nanofibers (ENFs)

**Fig. 9:**
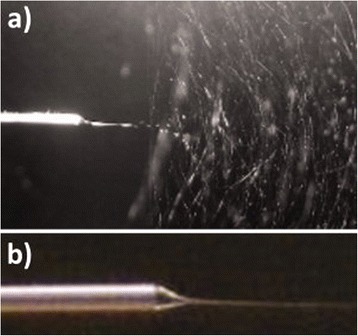
ENFs during the electrospinning process. **a** It shows the needle (*left*) and the fibers being electrospun towards the cathode (*right*). **b** Detailed view of the Taylor cone formed at the tip of the needle. Reprinted with permission from [88]. Copyright (2012) Wiley Periodicals Inc

A recent approach to create nanofibers is by the electrospinning method. This technique allows the production of synthetic nanofibers (polymeric, inorganic), denoted as electrospun nanofibers (ENFs). In 1995, Doshi and Reneker [86] reported for the first time the electrospinning process in a research article. At the beginning, this technique only consisted of a polymeric solution in a syringe with a needle, a high voltage source, and a metallic board as electrode, as shown in Fig. 8. The high voltage was applied to the polymeric solution with respect to the metallic board. This electrical field caused that an electrically charged polymeric jet was launched from the needle, forming the so-called Taylor cone (see Fig. 9a, b). The jet traveled in air, spinning into the Taylor cone, to the metallic board. The solvent of the solution was evaporated along the way, and ENFs were collected onto the board or the substrate to be coated [86–88].

In the last 15 years, the number of published electrospinning related works has experimented an almost exponential growth. This significant increase is due to its numerous advantages as simplicity, effectiveness, low-cost, quickness which allow the implementation of ENFs in a wide range of applications [89, 90]. Moreover, the ENFs structures present other strengths such as high surface area, small diameter, and specific physicochemical properties [91].

In this section, most used materials for the ENFs fabrication and novel ENFs with additional composites will be reported. Furthermore, a short list of the relevant applications of these types of nanofibers will be detailed, including some recent advances in diverse fields where ENFs are applied, as antibacterial coatings, water treatment, wound healing, and others.

#### Electrospun Nanofibers: Composition and Types

The composition of ENFs is very diverse, and their diameter can vary from 10 to 1000 nm or even more. Natural or synthetic polymers are used depending on the target or final application. Electrospun natural nanofibers of chitin, chitosan, hyaluronic acid, silk, cellulose, collagen, or others can be found in literature [92]. Regarding to the synthetic ones, a wide list of chemical compounds have been used by electrospinning: PA, PAA, poly(vinyl alcohol) (PVA), poly(ethylene glycol) (PEG), poly(ethylene oxide) (PEO), poly(ethylene-co-vinyl acetate) (PEVA), poly(acrylonitril) (PAN), poly(vinyl acetate) (PVAc), poly(lactic acid) (PLA), and more. A complete list of polymers which have been electrospun can be also found in [93].

Generally, these polymers are dissolved in an adequate solvent to obtain the electrospinning solution. The most used solvents are water, ethanol, n,n-dimethylformamide (DMF), and isopropanol, among others. Other important factors in the tailoring of the ENFs are the viscosity of the solution, flow rate, voltage level applied, and distance between syringe and collector. The precision of these parameters plays a key role in the ENFs fabrication [94].

**Fig. 10:**
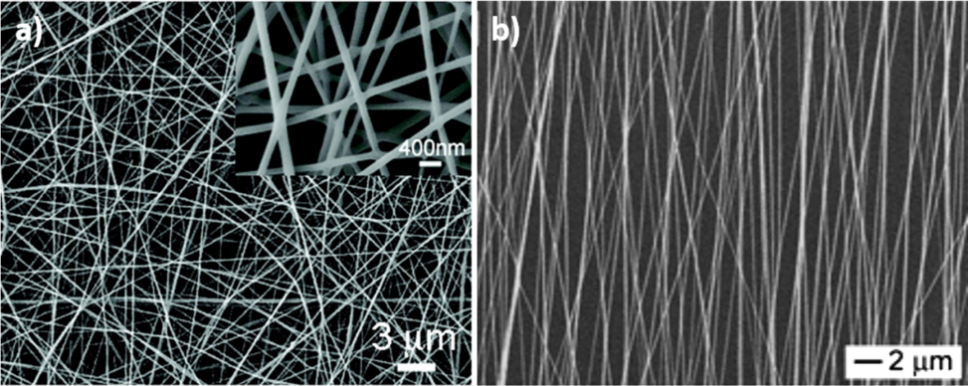
SEM images of electrospun nanofibers produced by electrospinning. **a** Nanofiber mat from random ENFs (*inset* shows the high-magnified SEM image); **b** aligned electrospun composite nanofibers. Reprinted (adapted) with permission from [96] and from [97], respectively. Copyright (2009) and Copyright (2003), American Chemical Society

ENFs are commonly classified in two types: randomly oriented ENFs and aligned ENFs. On one hand, the use of a simple electrospinning setup, as in Fig. 8, produces the first type of ENFs. The result is an electrospun nanoweb or nanofiber mat deposited onto the substrate (see Fig. 10a). On the other hand, aligned ENFs are mainly created by collecting the fibers over a rotating collector [95], generally a drum or a rotating disc. The alignment of the ENFs depends on the final applications (see Fig. 10b) [96, 97].

Nowadays, the polymeric solutions can be combined with the incorporation and use of organic and inorganic nanostructured materials or nanoparticles, obtaining novel electrospun composite nanofibers (ECNFs) and providing additional properties as reusability, high porosity, antibacterial activity, corrosion or chemical resistance, biomolecular affinity, etc [98]. Thus, the synthesis of ECNFs is performed by various methods. The simplest one consists of the dispersion of NPs or other nanostructures into the polymeric solution [99] before the fabrication process. A different approach is the coaxial electrospinning [100, 101], which combines two different stock solutions and produces ENFs with a core/sheath nanostructure. Other alternatives in the ECNFs fabrication are some specific post-treatments, such as thermal [102] or UV [103] curing, chemical functionalization [104], or binding [105], among others.

#### Electrospun Nanofibers: Applications

In the ENFs and ECNFs case, the applications depend strongly on the composition of the fibers and differ somewhat to the textile fiber applications. Thus, some of the most relevant applications of the ENFs and ECNFs are water treatment [106], antibacterial purposes [107], wound dressing [108], drug delivery [109], sensing [110, 111] and biosensing [112], tissue engineering [113], catalyst [114], microwave absorption [115], flame retardants [116], and other biomedical issues [117, 118].

As in the previous section, the recent advances in antibacterial coatings will be described. Furthermore, some relevant works for water treatment, wound healing and dressing, and other applications will be also presented because of their growing importance in the last years.

#### Antibacterial

The fabrication of antibacterial ENFs can be classified in several groups, depending on the used antibacterial agent. These active agents are incorporated in the fibers by mixing them with the polymeric solution before the electrospinning process, by coaxial electrospinning, by other chemical treatments or binding onto the ENFs surface. The most used active agents for the development of antibacterial ENFs are Ag NPs, antibiotics, triclosan, chlorhexidine, quaternary ammonium salts, chitosan, and metal oxide NPs.

The use of the silver as an antibacterial agent is well-known and has been applied to biomedicine and other fields. In the last years, Ag NPs have experimented a growing interest for their large surface area and better release of metal cations [119]. There are three main methods to incorporate Ag NPs into ENFs: by simple mixing with the polymer before electrospinning, by the in situ Ag NPs synthesis into the polymeric solution, and by the in situ synthesis of the Ag NPs after electrospinning in a post-treatment.

The first incorporation method of Ag NPs into the polymeric solution is the simplest, and it has been widely used. Recent works have reported the development of antibacterial ENFs thanks to this method, using polymers such as PVA [120], PVP [121], or poly(vinylidene fluoride) (PVDF) [122].

The second method is based on the in situ Ag NPs synthesis into the electrospinning solution. For this purpose, silver nitrate (AgNO_3_) is added to the polymeric solution. Here, the polymer acts as encapsulating agent for the silver cations. Next, a reducing agent is incorporated to the solution, thus forming Ag NPs. In some cases, the own solvent acts as a reducing agent. Examples of ECNFs fabricated with this method have been published by Le et al. [123] and Yang et al. [124] using PAN/AgNO_3_/DMF mixing solution. In addition, other studies have successfully developed ECNFs from nylon/AgNO_3_/formic acid [125] and PVA/AgNO_3_/chitosan [126]. The main advantage of this method is the fact that it does not need to pre-synthetize the Ag NPs, and moreover, the ECNFS do not require further treatments.

**Fig. 11:**
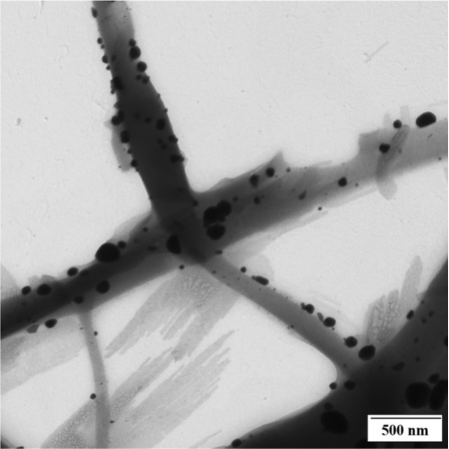
TEM image of the Ag-loaded electrospun nanofibers. Ag NPs can be observed onto the ENFs surface. Reprinted with permission from [88]. Copyright (2012) Wiley Periodicals Inc

**Fig. 12:**
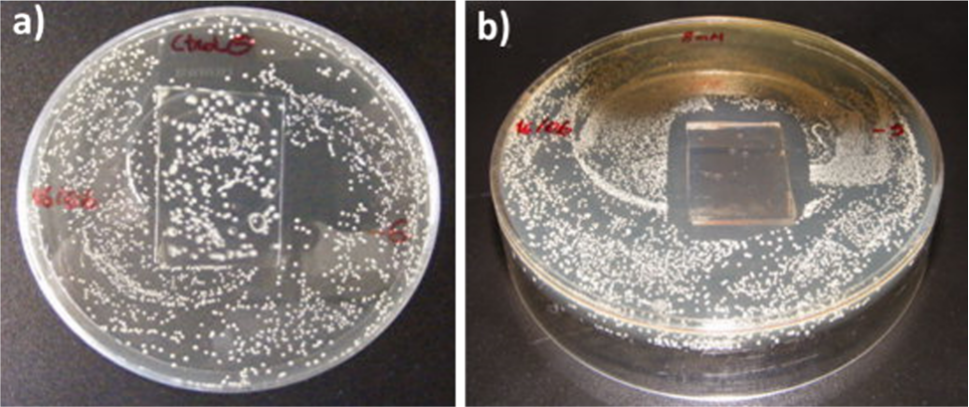
Bacteriological cultures of *Lactobacillus plantarum* after 24 h with two different samples. **a** Reference sample of PAA electrospun nanofiber mat with no silver nanoparticles; **b** silver-loaded PAA electrospun fiber mat. Substrate: glass slides. Reprinted with permission from [88]. Copyright (2012) Wiley Periodicals Inc

The third method to obtain ENFs with Ag NPs is based on diverse treatments after the electrospinning process. In a first step, the ECNFs are fabricated. Then, a further thermal, UV, or other chemical treatments are performed. Thus, PVA/regenerated silk fibroin ENFs have been heated at 155 °C for 5 min or treated with UV for 3 h [127], PLA fibers at 80 °C for 48 h in a hydrogen atmosphere [128], or PAN ENFs at 160 °C [129] to allow the transition of Ag cations to metallic silver. Nevertheless, such post-treatments are not as effective as the Ag NPs synthesis in the polymeric solution [129]. However, in a recent separate study reported by Rivero et al. [88], PAA/cyclodextrin nanofibers were thermally treated and then processed by dip-coating as follows. In a first step, nanofibers were immersed in an AgNO_3_ solution, charging the ENFs surfaces with Ag cations. Next, ENFs were immersed in dimethylamine borane, leading to the reduction of Ag cations to Ag NPs onto the ENF mat surface. A TEM image of the Ag NP-loaded ENFs and antibacterial test results from this work are shown in Figs. 11 and 12, respectively.

Not only Ag NPs exhibit antibacterial properties but also ZnO and TiO_2_ NPs, as it was previously commented. These NPs present excellent antibacterial behavior after or during UV illumination [130, 131]. However, the necessity of a UV radiation makes them less attractive from the antibacterial point of view.

In the antibiotics case, the amount of drug is adjusted in the electrospinning solution. The concentration of each antibiotic is different for each solution. Thus, Kenawy et al. [132] used blends of PLA and PEVA containing 5 wt.% tetracycline hydrochloride. In other works, the quantities are 30 wt.% of Moxi in coPLA [133] and 7.5 wt.% mupirocin in PLA fibers [134], respectively. Although the antibiotic-polymer mixture has advantages as simplicity and flexibility in the amount of the drug loaded, the ENFs tend to leach out the active agent too fast into the solution, which is known as burst release phenomenon. To avoid this and provide a well-controlled release, some approaches have been developed using coaxial electrospinning, fabricating core/sheath structures. For example, antibiotics as ampicillin [135], tetracycline hydrochloride [136], or gentamycin [137] have been successfully encapsulated in diverse ENFs.

**Fig. 13:**
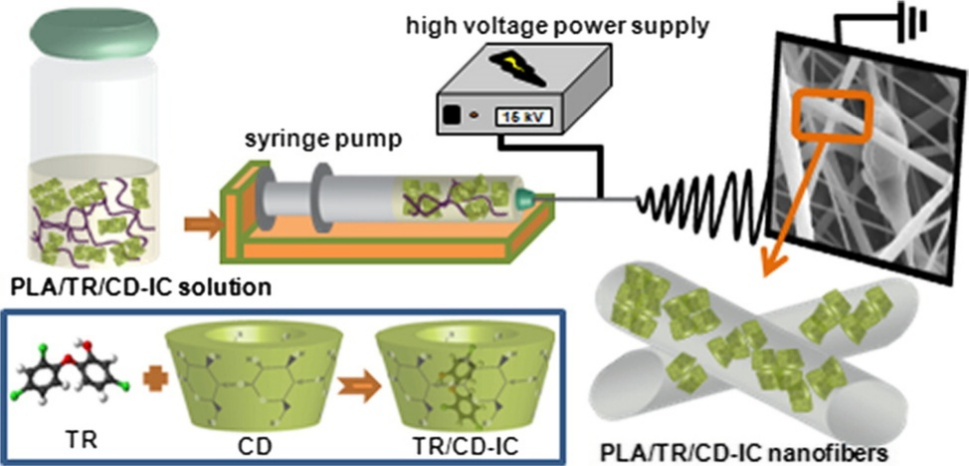
Schematic of the fabrication ECNFs process of triclosan/cyclodextrin-induced complexes (TR/CD-IC) with PLA performed by Kayazi et al. Reprinted with permission from [140]. Copyright (2013) American Chemical Society

**Fig. 14:**
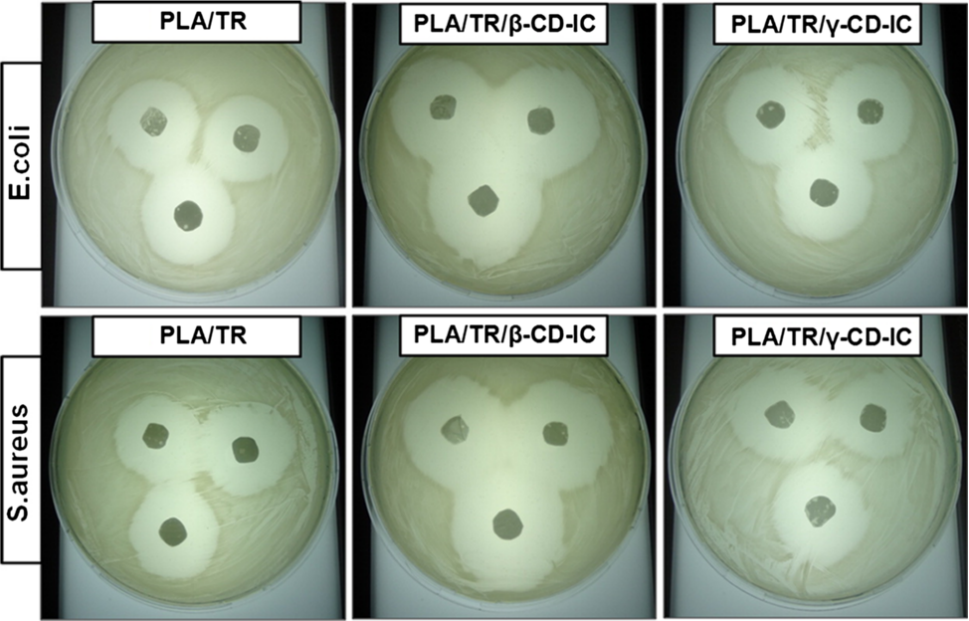
Antibacterial results of the ECNFs with and without different types of CD. The bacterial inhibition growth is higher for the PLA/TR/β-CD-IC nanofibers. Reprinted with permission from [140]. Copyright (2013) American Chemical Society

Other biocide compounds have been also mixed into electrospinning solution as chlorhexidine [138] in cellulose acetate or amine groups [139] in PAN. In another approach reported by Kayaci et al. [140], triclosan (TR) have been complexed with cyclodextrin (CD) and mixed with PLA. Thanks to the complexation, the ECNFs showed an increase in their antibacterial activity for *E. coli* and *S. aureus*. A schematic of the ECNFs fabrication and their antibacterial results are shown in Figs. 13 and 14, respectively.

Chitosan is another natural substance which has an inherent antibacterial ability, and therefore, it has been also used to fabricate antibacterial ENFs. Due to its weak mechanical properties, chitosan has been mixed with PVA, PLA, PEO, or PA to develop stronger ECNFs. More information about the use of chitosan in antibacterial ENFs can be found in literature [141].

#### Wound Dressing

Selected wound dressing materials are required with special attention for the wound healing process. Among the overall compounds of great importance, Ag NPs are used as antimicrobial in hydrocolloids, alginates, and hydrofibrous materials fabricated by electrospinning [142]. For example, ECNFs bandages have been developed using iodines encapsulated into PVP and PEO/PVP ENFs [143]. Other relevant works focused on the preparation of wound dressing mats by electrospinning have been presented by Hong [144] and Rujitanaroj et al. [145].

Hong reported a novel wound dressing material based on PVA/AgNO_3_ nanofiber mats. In a first stage, PVA and AgNO_3_ were mixed and launched by electrospinning. After that, the resultant nanofiber webs were treated by heat or UV radiation. The study concluded that the only heat-treated electrospun PVA/AgNO_3_ fiber web was a good material as wound dressings because it had structural stability in moisture environment as well as excellent antimicrobial ability and quick and continuous release of the effectiveness [144].

Rujinaroj et al. developed successful gelatin fiber mats containing Ag NPs. The stability of the fiber mats were tested in an aqueous solution, the release of the Ag cations was studied in a simulated body fluid (pH 7.4) at skin temperature (32 °C) and physiologic temperature (37 °C), and the antibacterial activity was analyzed [145].

#### Water Treatment

Water pollution is a high problem nowadays, and the wastewater and pollutants may impact dramatically in the aquatic life and humans. To overcome this, there are some techniques which are developing interesting solutions. One of these processes is the photocatalysis, which allows the photodegradation of some pollutants present in water. Recently, new advances in ENFs with photocatalytic nanostructures have been reported. In a study, Panthi et al. [114] developed ECNFs using PAN and in situ synthesized CoS NPs. Their photocatalytic activity was analyzed, and results showed excellent behavior for photodegradation of methylene blue and methyl red dyes under solar light radiation. In other example, Yu et al. [146] fabricated ECNFs based on PAN and Ag_3_PO_4_ and analyzed their photocatalytic activity under visible radiation. They demonstrated that the ECNFs exhibit higher photocatalytic performances than pure Ag_3_PO_4_ powder. They indicated that the combination of particle-polymer for the ECNFs preparation may be adopted for more applications. Furthermore, TiO_2_ and ZnO have well-known photocatalytic properties. Both metal oxides have been also used to fabricate ECNFs for wastewater treatment [147, 148].

#### Other Applications

Between other important applications are tissue engineering and drug delivery. In tissue engineering, the ECNF material must be biocompatible. For example, poly(l-lactide-co-ε-caprolactone) has been used to fabricate electrospun tubular scaffolds for tissue engineered vascular grafts [149]. Another vascular application was reported by He et al. [150]. They demonstrated in an in vitro study that the incorporation of electrospun collagen-blended poly(l-lactic acid)-co-poly(ε-caprolactone) scaffolds enhances human coronary artery endothelial cell viability, spreading, and attachment, while also preserving the endothelial cell phenotype . Other emergent developments in tissue engineering based on electrospinning are focused on potential scaffolds for ligaments [151], tendons [152], and bones [153], thanks to the alignment and the mechanical properties of the ECNFs.

Regarding drug delivery applications, ENFs with high porosity allow drug loadings and have the ability to overcome mass transfer limitations associated with other polymeric systems. Thus, in some works, drug is located by the absorption onto the surface of the ENFs and then their efficacy was analyzed in in vivo studies [154]. Also in the drug delivery field, the targeted and controlled delivery of anticancer drugs is being one of the widest areas of research now [155, 156].

Another recent application based on ECNFs is their use as flame retardants. Wu et al. [157] reported novel ECNFs composed of nylon 6 with two additives: montmorillorite nanoclay and a non-halogenated flame retardant based on organic phosphinates. The three components were mixed, and the ECNFs were performed and analyzed. Although the termogravimetric analysis presented worse results, the loss mass rate of the samples was overall decreased, and the additives played a key role in reducing flammability. In other study, Selvakumar et al. [116] developed ENFs of PA with boronic acid NPs onto a cotton substrate. With a very low concentration of these resultant ECNFs, they increased the flame-spreading time in 80 %, obtaining excellent fire protection results.

Finally, a summary of some of the most relevant works and their applications are summarized in Table 4, showing in each case, the polymeric precursors and the active agents used to the ECNFs fabrication.

**Table 4:** Summary of some of the most relevant works classified by applications, indicating the polymer precursors and the loaded active agents for the electrospun nanofibers fabrication

Antibacterial applications		
Antibacterial agent	Polymeric precursor	Antibacterial tests
Ag NPs (from AgNO_3_ reduction)	Poly(acrylic acid) (PAA)/cyclodextrin	*L. plantarum*, 2012 [88]
Ag NPs (from aqueous solution)	Poly(vinyl alcohol) (PVA)	*S. aureus* and *E. coli*, 2010 [107]; 2011 [120]
Ag NPs (by seed mediate growth method)	Poly(vinyl pyrrolidone) (PVP)	*S. aureus, K. pneumoniae*, and *E. coli*, 2011 [121]
Ag NPs (in ethanol solution)	Poly(vinylidene fluoride) (PVDF)	*S. aureus* and *K. pneumoniae,* 2010 [122]
Ag NPs (from AgNO_3_ reduction)	Poly(vinyl alcohol) (PVA) and chitosan	*E. coli*, 2012 [126]
Ag NPs (from AgNO_3_ reduction)	Polyvinyl alcohol (PVA)/regenerated silk fibroin	*S. aureus* and *E. coli*, 2011 [127]
Ag NPs (from AgNO_3_ reduction)	Poly(acrylonitrile) (PAN)	Not tested, 2005, 2003 [123]; [124]. *E. coli*, 2012 [129]
Ag NPs (from AgNO_3_ reduction)	Nylon 6	*B. cereus* and *E. coli*, 2011 [125]
Quaternary ammonium salts	Diblock copolymers with polyacrylates	*S. aureus* and *E. coli*, 2008 [139]
Triclosan	Poly(lactic acid) (PLA), cyclodextrin	*S. aureus* and *E. coli*, 2013 [140]
Chlorhexidine	Cellulose acetate	*E. coli* and *S. epidermidis*, 2008 [138]
Other Applications		
Active agent	Polymeric precursor	Application
Mupirocin (antibiotic)	Poly-l-lactic acid	Drug release, 2008 [134]
Tetracycline hydrochloride (antibiotic)	Poly(lactic acid) (PLA), poly(ethylene-co-vinyl acetate) (PEVA); poly(l-lactid-co-ε-caprolactone)	Drug release, 2002 [132], 2009 [136]
Fluoroquinolone antibiotics	Poly(l-lactide-co-d,l-lactide) and coPLA/poly(ethylene glycol)	Drug release, 2012 [133]
Ampicillin (antibiotic)	Poly(methyl methacrylate)-nylon 6	Drug release, 2013 [135]
Gentamycin sulfate (antibiotic)	Polycaprolactone	Drug release, 2006 [137]
Iodines	Poly(vinyl pyrrolidone) (PVP)	Wound dressing, 2007 [143]
Ag NPs	Gelatin; poly(vinyl alcohol)	Wound dressing mats, 2007, 2008 [144, 145]
CoS NPs; Ag_3_PO_4_	Poly(acrylonitrile) (PAN)	Water treatment, photocatalyst, 2013, [114, 146]
CdO, ZnO, TiO_2_ (photocatalytic)	Poly(vinyl alcohol)	Water treatment, 2012 [147], 2010 [148]
Collagen; Cell adhesive peptides	Poly(L-lactic acid)-co-poly(ε-caprolactone); poly(b,l-lactic-co-glycolic acid) (PLGA)	Tissue engineering, 2006 [149], 2005 [150]
Hydroxyapatite, PLGA	Poly-l-lactic acid; PLGA	Tendons/ligaments/bones tissue engineering, 2006 [152], 2007 [153]
Boronic acid NPs	Polyamide 6	Flame retardant, 2012 [116], 2014 [157]

## Conclusions

Nanoparticles are a new promising kind of materials for functionalization of fibers and textiles. Their outstanding properties derived from their size and extremely high specific surface make them one of the most useful additives to provide of additional functionality to fibers and textiles. Their low cost, lower than other nanostructures, make them suitable for industrial applications. Although there are still some problems with their incorporation to commercial applications, there are numerous significant scientific research works that report their suitability for high-value functional textile applications.

The addition of nanoparticles to traditional synthetic fibers is usually performed using well-known techniques such as pad-dry-cure processes; nevertheless, the appropriate surface chemistry of nanoparticles to achieve a good adhesion or even chemical bonding to the fibers is where the technological challenge is. Other techniques such as corona treatments and in situ synthesis of the nanoparticles have been successfully used as alternative functionalization techniques with very good results. Regarding to the scientific bibliography, the antibacterial functionality is the most demanded application for nanoparticles into textile fibers, due to their use in the elaboration of clothes, but there are other properties such as flammability, light absorption, and wettability that can be dramatically affected by the presence of the appropriate nanoparticles.

Furthermore, there are special nonwoven micro and nano textiles which can be obtained using the electrospinning technique. Their ultrathin diameter and the good control of their internal structure, density, and porosity make them ideal for very specific high technology applications. Their controllable composition and hosting properties for different nanoparticles allow to create breathable fiber mats with antibacterial properties which can help in the healing of wounded skin. But such porous membranes have been also investigated for filters and water treatment applications and other kinds of tunable membranes. The apparition of all these functional artificial fibers and smart fabrics mean undoubtedly the new revolution for the textile industry.
